# Electrical aligned polyurethane nerve guidance conduit modulates macrophage polarization and facilitates immunoregulatory peripheral nerve regeneration

**DOI:** 10.1186/s12951-024-02507-3

**Published:** 2024-05-12

**Authors:** Yiting Sun, Yinglong Zhang, Yibo Guo, Dongming He, Wanlin Xu, Wei Fang, Chenping Zhang, Yi Zuo, Zhen Zhang

**Affiliations:** 1 Department of Oral & Maxillofacial-Head & Neck Oncology, Shanghai Ninth People’s Hospital, Shanghai Jiao Tong University School of Medicine, College of Stomatology, Shanghai Jiao Tong University, National Center for Stomatology, National Clinical Research Center for Oral Diseases, Shanghai Key Laboratory of Stomatology, Shanghai, 200011 China; 2 Research Center for Nano-Biomaterials, Analytical and Testing Center, Sichuan University, Chengdu, 610064 China; 3 Department of Oral & Cranio-Maxillofacial Surgery, Shanghai Ninth People’s Hospital, Shanghai Jiao Tong University School of Medicine, College of Stomatology, Shanghai Jiao Tong University, National Center for Stomatology, National Clinical Research Center for Oral Diseases, Shanghai Key Laboratory of Stomatology, Shanghai, 200011 China; 4 MOE Key Laboratory of Low-Grade Energy, Utilization Technologies and Systems, CQU-NUS Renewable, Energy Materials & Devices Joint Laboratory, School of Energy & Power Engineering, Chongqing University, Chongqing, 400044 China; 5 Department of Oral and Maxillofacial Surgery, Zhongshan Hospital, Fudan University, Shanghai, 200032 China; 6 Shanghai Stomatological Hospital & School of Stomatology, Fudan University, Shanghai, 200032 China

**Keywords:** Nerve regeneration, Macrophage polarization, Polyurethane, Aligned nanofibers, Nerve guidance conduit

## Abstract

Biomaterials can modulate the local immune microenvironments to promote peripheral nerve regeneration. Inspired by the spatial orderly distribution and endogenous electric field of nerve fibers, we aimed to investigate the synergistic effects of electrical and topological cues on immune microenvironments of peripheral nerve regeneration. Nerve guidance conduits (NGCs) with aligned electrospun nanofibers were fabricated using a polyurethane copolymer containing a conductive aniline trimer and degradable L-lysine (PUAT). In vitro experiments showed that the aligned PUAT (A-PUAT) membranes promoted the recruitment of macrophages and induced their polarization towards the pro-healing M2 phenotype, which subsequently facilitated the migration and myelination of Schwann cells. Furthermore, NGCs fabricated from A-PUAT increased the proportion of pro-healing macrophages and improved peripheral nerve regeneration in a rat model of sciatic nerve injury. In conclusion, this study demonstrated the potential application of NGCs in peripheral nerve regeneration from an immunomodulatory perspective and revealed A-PUAT as a clinically-actionable strategy for peripheral nerve injury.

## Introduction

Peripheral nerve injury (PNI) caused by trauma, surgical intervention, or infection is a common clinical condition that affects over one million patients worldwide annually [1, 2]. It often results in permanent damage to sensory perception and motor function, which reduces the quality of life of patients [3]. Although peripheral nerves, unlike the central nervous system, can spontaneously regenerate after injury, their functional recovery is usually poor, especially in long gaps [4].

Autologous nerve grafting remains the gold standard for bridging peripheral nerve gaps. However, it is limited by mismatches between the donor nerves and the recipient sites, donor site morbidity, limited supply of available grafts, and the need for multiple surgeries [5, 6]. Artificial nerve grafts may be an alternative to autologous nerve grafts [4, 7]. In recent years, various types of artificial nerve grafts, mostly in the form of nerve guidance conduits (NGCs), have been designed, fabricated, and tested in vivo [4, 7–10]. The materials for NGCs should have biocompatibility, low immunogenicity, mechanical support to keep the nerve gaps, suitable elasticity, appropriate degradation behavior, and electrical conductivity [4, 7].

The performance of an implanted biomaterial depends on the host’s reaction following implantation. NGCs made from biomaterials are promising alternatives for peripheral nerve regeneration [4, 7]. Previous studies have mainly focused on optimizing the nerve regenerative capacity, such as the proliferation, migration, and differentiation of peripheral nerve cells. However, only a few studies have investigated the effects of nerve regeneration on immune responses. The immune system and the peripheral nervous system maintain extensive interactions. NGCs with favorable immunomodulatory properties may be a key tool for tissue regeneration [11–13].

Macrophages are the first immune cells that appear at an injured site after nerve injury or implantation, and they are responsible for modulating the microenvironment and orchestrating nerve regeneration [13, 14]. They can tactically shift between the classically activated phenotype (M1, pro-inflammatory macrophages) and the alternatively activated phenotype (M2, pro-healing macrophages) under different conditions [11–15]. The high plasticity of macrophages makes them a potential target in nerve regeneration. Studies have proved that topographic cues could affect macrophage phenotype through modulating the cytoskeleton. M2 macrophages demonstrated elongated shape than M1 macrophages [16]. Oriented electrospinning nanofibers could modulate the shape of macrophages to an elongated state to promote M2 polarization and reduce the secretion of pro-inflammatory cytokines [17]. Moreover, small pore sizes would reduce the macrophage response to inflammatory stimuli [18]. Similarly, electrical stimulation, one of the physical stimuli, has been shown to regulate the polarization of macrophages and thus the repair of other tissues. Piezoelectricity of BT/Ti (poled) scaffolds activated oxidative phosphorylation and ATP synthesis to promote M2 polarization of macrophages and stimulated immunoregulatory osteogenesis [19]. Also, electrical stimulation provided from conductive hydrogels regulated macrophage polarization and thus promoted wound healing [20]. Therefore, it is a feasible strategy to regulate macrophages through biomaterials to promote nerve regeneration.

Polyurethane is one of the most widely used synthetic elastic polymers in tissue engineering [21–23]. In our previous study, a new electroactive polyurethane containing an aniline trimer and L-lysine (PUAT) was designed, fabricated, and characterized [22]. Owing to its excellent properties of biocompatibility, mechanics, degradation, and electrical conductivity, PUAT may be a promising material for NGCs.

In this study, inspired by the spatial orderly distribution and endogenous electric field of nerve fibers, we fabricated a conductive NGC with aligned topography and electroactive aniline trimer-based polyurethane (PUAT). We examined macrophage polarization induced by PUAT membranes with random or aligned nanofibers and explored their association with the migration and differentiation of Schwann cells (SCs). Furthermore, NGCs with random or aligned nanofibers, respectively, were grafted in a rat model of sciatic nerve injury, and their effects on macrophage polarization and nerve regeneration in vivo were investigated.

## Materials and methods

### Synthesis of PUAT membranes and NGCs

Poly(ε-caprolactone) diol (PCL diol, Mn = 2000) was purchased from Beijing J&K Scientific Ltd. Isophorone diisocyanate (IPDI), L-lysine, aniline, and p-phenylenediamine were purchased from Shanghai Aladdin Co. Ltd. Hydrochloric acid (HCl), ammonium persulfate, dimethyl sulfoxide (DMSO), N, N-dimethylformamide, N-methylformamide, methanol, and aqueous ammonia were obtained from Chengdu Kelong Co. Ltd. All chemical reagents used in this study were of analytical grade.

Amine capped aniline trimer (AT) was first synthesized according to our previous study [24]. The PUAT copolymers were synthesized in a three-necked flask under an nitrogen (N_2_) atmosphere, with PCL as the polyester soft segment and L-lysine and AT as the chain extenders as reported previously [22]. Firstly, IPDI, PCL, and stannous salt were used as hard segments, soft segments, and the catalyst respectively to synthesis the prepolymer in a three-neck flask withN_2_ atmosphere. After stirring at 70 °C for hours pre-polymerization, lysine was added into the solution and reacted for another 2 h at 50 °C to extend the chain of copolymer. Then, AT was added and stirred slowly till the reaction was terminated. The molar ratio of PCL/ IPDI/ L-lysine/ AT = 2:4:1:1. The copolymers were purified and washed by methanol and deionized water, respectively. Finally, the obtained products were vacuum-dried for 5 days to eliminate solvent adequately.

The PUAT membranes with different topological structures were fabricated by electrospinning technique. The PUAT copolymers were dissolved in hexafluoroisopropanol (HFIP) at a concentration of 15% (w/v) to obtain the spinning solution. For electrospinning, the voltage between the needle and the receiver was 12 kV, the ambient humidity was maintained at 40% and the flow rate of the solution was 0.5 mL/h. A rotating cylinder with the rotating speed of 1000 rpm and a plate receiver were used to obtain the PUAT membrane in an aligned or random orientation, respectively. Then, the PUAT fibrous membrane was rolled up into a conduit using a custom steel wire mold with the diameter of 1.5 mm and the length of 20 cm. Whereafter, the fabricated conduits were immersed in the deionized water for 3 days and then placed in a freeze-drying oven for another 3 days. The above procedures were repeated 2 times to fix the size of conduits. To compare the effects of conductive polymer and non-conductive polymer on the polarization of macrophages, we also constructed PCL membranes and conduits with random topography. PCL membranes and conduits were prepared by the same method as above. The samples were named as PCL, PUAT with randomly orientated nanofibers (R-PUAT), and PUAT with aligned nanofibers (A-PUAT).

### Characterization of the membranes

The chemical composition of different membranes was analyzed by FT-IR (Nicolet 8700, Thermo, USA). Surface morphology of the membranes was analyzed by scanning electron microscopy (SEM; JSM-6700 F, JEOL, Japan) and an atomic force microscope (AFM; Multimode & Dimension 3100, Varian, USA). The surface hydrophilicity evaluation by water contact angle measurement was determined by a contact angle goniometer (Dataphysics OCA 35, Germany) using the sessile drop method. The mechanical properties of the membranes were evaluated by a universal testing machine (AG-IC 50KN, SHIMADZU, Japan). The samples were cut into dumbbells with a size of 4 mm × 50 mm. The crosshead speed was set at 15 mm/min with a load capacity of 50 N. The tensile strength, breaking elongation, and modulus data were obtained. The electroactivity of PUAT copolymer was investigated by the cyclic voltammetry (CV) on an electrochemical workstation (CHI 660E, China) with a scanning rate of 10 mV/s in 1 M HCl solution. In the electrode system, the graphite rod was immersed in the PUAT solution and dried in the oven. The PUAT copolymer was coated on the graphite rod and employed as working electrode. A platinum plate, and an Ag/AgCl were used as counter and reference electrodes, respectively.

### In vitro experiments

#### Cell culture

The rat Schwann cells (RSC96) and the murine macrophage cells (RAW264.7) were purchased from Typical Culture Preservation Commission Cell Bank, Chinese Academy of Sciences (Shanghai, China). RSC96 and RAW264.7 cells were cultured in Dulbecco’s Modified Eagle Medium (DMEM; Gibco) containing 10% fetal bovine serum and 1% penicillin/streptomycin and maintained under a humidified atmosphere of 5% CO_2_ at 37℃.

#### Cell adhesion and viability

RAW264.7 cells (2 × 10^4^ cells) were cultured in 24-well plates with PCL membranes, R-PUAT membranes and A-PUAT membranes. After 3 days, cells were fixed with 2.5% glutaraldehyde and dehydrated through ethanol. SEM was utilized for detecting the morphology of macrophages.

To investigate the effect of membranes on cell viability, RAW264.7 cells were seeded on different membranes, while those seeded on plates without membranes were considered as controls. At indicated time points, Cell Counting Kit-8 (CCK-8; Sigma, USA) was used according to the manufacturer’s introductions.

#### Gene expression and RNA-sequencing analyses of RAW264.7 cells

To determine the transcription levels of inducible nitric oxide synthase (Nos2) and arginase 1 (Arg-1) of Raw264.7 cells cultured on different substrates, lipopolysaccharide (LPS, 100ng/mL)-treated RAW264.7 cells were subjected to quantitative real-time polymerase chain reaction (qRT-PCR) with the SYBR Premix Ex Taq II PCR Kit (Takara, Japan) after culturing for 1 and 3 days.

For RNA-sequencing analysis, total RNA was extracted from the cell samples using TRIzol reagent (Invitrogen, USA), according to the manufacturer’s instructions. cDNA libraries were then constructed for pooled RNA samples using the VAHTSTM Total RNA-Seq (H/M/R) Library Prep Kit (VAHTSTM, China). Differentially expressed genes (DEGs) were identified using TopHat and Cufflinks, and their expression levels were determined by the fragments per kilobase of transcript per million mapped reads method. The DESeq algorithm was used to screen the DEGs between groups. The biological functions of the DEGs were then reviewed by Gene Ontology (GO) enrichment analysis (http://www.geneontology.org/).

#### Enzyme-linked immunosorbent assay (ELISA)

The supernatants of LPS-treated RAW264.7 cells which were cultured on different membranes were collected after 24 h and 72 h.The levels of the released cytokines tumor necrosis factor-alpha (TNF-α) and interleukin 10 (IL-10) were measured by ELISA kits (Thermo Fisher Scientific, USA) according to the manufacturer’s instructions.

#### Immunofluorescence staining

After 3 days of culture, RAW264.7 cells on different membranes were fixed in 4% paraformaldehyde (PFA) for 10 min, washed with PBS, permeabilized with 0.3% Triton, and blocked by 5% BSA. Then samples were cultured overnight at 4 ℃ with primary antibodies to detect CD206 (1:500, 24595, CST). Samples were then incubated with appropriate secondary antibodies and stained with FITC-phalloidin for 1 h at 37 °C and counterstained in DAPI for 10 min at room temperature. Confocal laser scanning microscopy (CLSM) was used to observe the cell structure.

#### Flow cytometry

Flow cytometry was performed to determine the phenotypes of macrophages by examining the expression levels of the M1 marker CD80 and the M2 marker CD206. After seeding on different membranes for 72 h, LPS-treated RAW264.7 cells were digested, rinsed twice with PBS, and then incubated with FITC-conjugated anti-mouse CD80 antibody (1:50; BioLegend) and FITC-conjugated anti-mouse CD206 antibody (1:400; BioLegend) respectively. The labeled cells were then rinsed twice with PBS and examined by a flow cytometer (BD Accuri C6, BD Biosciences, USA). FlowJo 10.0 software was used for the quantitative analysis.

#### The effects of polarized macrophages on the proliferation, migration, and myelination of RSC96 cells

To evaluate the effects of macrophage polarization on the proliferation, migration, and myelination of RSC96 cells, the supernatants of LPS-treated RAW264.7 cells which were seeded on different membranes for 72 h were collected as the conditioned medium (CM). According to relative studies [13, 17, 25], RSC96 cells were seeded in 96-well plates and then treated with a mixture of DMEM and CM at a ratio of 1:1. After culturing for 1 and 3 days, respectively, the CCK-8 assay was performed to analyze the proliferation of RSC96 cells.

Wound healing assays and Transwell assays were performed to examine the effects of polarized macrophages on the migration of RSC96 cells. In the wound healing assay, RSC96 cells at a concentration of 2 × 10^4^ cells/well were seeded in 12-well plates. The cell monolayer was scratched with a 200-µL pipette tip, and the scraped cells were washed off with PBS. Then, 1 mL of DMEM supplemented with CM from different groups was added to each well. The migration of RSC96 cells was observed under an inverted fluorescence microscope (IX71, Olympus, Japan) at 0, 12, and 24 h.

Boyden chambers were used for the Transwell assay. RSC96 cells were added to the upper chambers of 24-well Transwell plates (8-µm pore size, Costar, USA), while membranes seeded with LPS-treated RAW264.7 cells were placed in the bottom chambers of the plates. After incubation at 37 °C for 24 h, RSC were washed with PBS, fixed in 4% PFA, and stained with 0.1% crystal violet. The non-migrated cells in the upper chambers were wiped off with a cotton swab. Cells outside of the filters were counted and imaged by an inverted optical microscope (IX71, Olympus, Japan).

The effect of polarized macrophages on myelination of RSC was determined through qRT-PCR and immunofluorescence staining. RSC were cultured with different CM for 3 days. The transcription levels of peripheral myeline protein 22 (Pmp22), nerve growth factor (Ngf), and neural cell adhesion molecule 1 (Ncam1) were determined.

As for immunofluorescence staining, all samples were fixed with 4% PFA, permeabilized with 0.3% Triton-X, and blocked by 5% BSA. Cells were then cultured with primary antibodies MPZ (1:500, 10572-1-AP, Proteintech) overnight at 4 ℃. Samples were then incubated with appropriate secondary antibodies and DAPI. Finally, cells were observed under a confocal microscope.

### In vivo studies

#### Ethics statement

All animal experiments were approved by the Animal Experiment Ethics Committee of the Shanghai Ninth People’s Hospital affiliated with Shanghai Jiao tong University (Approval number: SH9H-2021-A48-SB). The study design was in accordance with the Animal Research: Reporting of In Vivo Experiments (ARRIVE) Guidelines.

#### Surgical procedure

Male Sprague-Dawley (SD) rats (weighing 250–300g) were used to investigate the effects of different NGCs on sciatic nerve repair. In brief, SD rats were randomly divided into four groups: PCL, R-PUAT, A-PUAT, and Auto. A 10-mm-long sciatic nerve defect was created in the left thigh of each rat. PCL, R-PUAT, and A-PUAT NGCs were grafted to bridge the nerve gap using 8 − 0 nylon sutures. In the Auto group, a 10-mm nerve segment was excised and replaced with an autograft (180° reversal of the excised segment). The wound was then closed. All animals were housed and fed routinely until they were euthanized at the designated time points.

#### Walking track analysis

Walking track analysis was performed to evaluate the sciatic nerve functional recovery 12 weeks after the operation. Each rat was tested in one confined walkway (10 cm × 100 cm covered with white paper) and the Sciatic Functional Index (SFI) value was analyzed through their ink footprints. The SFI value ranges between − 100 and 0, where − 100 indicates total loss of function, while 0 means normal nerve function.

#### Electrophysiological evaluation

At 12 weeks post-operation, individual rats were subjected to anesthesia, and the injured sciatic nerves were re-exposed for electrophysiological evaluation. An electrical stimulation was applied to the nerve trunk at the proximal end of the graft site, and a recording electrode was placed in the gastrocnemius belly. Then, the compound muscle action potentials (CMAP) and the time to deflection (latency) were recorded by an 8-channel physiological signal recorder (RM-6280 C, Chengdu Instrument Factory, China).

#### Evaluation of gastrocnemius muscle

The gastrocnemius muscles of both the experimental and control sides of each rat were dissected and weighed. The moist muscle weight ratio was calculated as follows: Moist weight of the experimental side / Moist weight of the control side × 100%. Next, the muscles were fixed, paraffin-embedded and sectioned for Masson’s staining. The percentage of collagen was also evaluated.

#### Immunofluorescent, histological, morphological, and immunohistochemical analyses of repaired nerves

For investigating the polarized macrophage and migrated SCs surrounding nerve defects, individual animals were euthanized at 7 and 14 days post injury and their left nerves were collected for immunofluorescence staining. Briefly, after fixation, embedded and sectioned, different slices were stained with CD68 (1:100, ab31630, Abcam), iNOS (1:70, ab15323, Abcam), CD206 (1:100, ab64693, Abcam), and S100β (1:100, ab52642, Abcam).

The rats were euthanized at 12 weeks post-operation, and the regenerated nerves were isolated for the following analyses. For the histological analysis, the transverse sections of the middle portion of the regenerated nerves were fixed, dehydrated, embedded in paraffin, and stained with hematoxylin and eosin (H&E) or Luxol fast blue (LFB). Images were observed under a light microscope (DM3000, Leica, Germany). The percentage of LFB-positive area was determined. For transmission electron microscopy (TEM), the samples were fixed in 2.5% glutaraldehyde (Solarbio, China) and stained with lead citrate and uranyl acetate. Subsequently, the morphology of the regenerative nerves was analyzed by TEM (HT7700 Exalens,  HITACHI, Japan). For multiplex immunofluorescence staining, a TSAPLus kit (G1236, Servicebio) was used according to the manufacturer’s instructions to avoid non-specific staining of antibodies from the same species. Primary antibodies of anti-PMP22 (1:200, sc-515199, Santa Cruz), anti-NCAM (1:1000, ab220360, Abcam) and anti-PGP9.5 (1:1000, ab10404, Abcam) were used. All tissue samples were then stained with goat anti-mouse IgG secondary antibody (1:200, Sigma) or goat anti-rabbit IgG secondary antibody (1:200, Sigma). Cell nuclei were counterstained with DAPI. The Panoramic Scanner with Panoramic DESK, P-MIDI, and P250 (3D HISTECH, Hungary) was used for observation.

### Statistical analysis

All statistical analyses were performed using GraphPad Prism 8.0 (GraphPad Software Inc., USA). Quantitative data represented the results of at least three independent experiments and were expressed as the mean ± standard deviation. Single comparison was performed using an unpaired Student’s *t*-test. Multiple comparisons were performed using one-way analysis of variance and Tukey’s post-hoc test. Multivariant comparisons were carried out using two-way analysis of variance with Tukey’s post-hoc test. The minimum significance levels were set at *p < 0.05, **p < 0.01, and ***p < 0.001.

## Results

### Characterization of the membranes

**Fig. 1:**
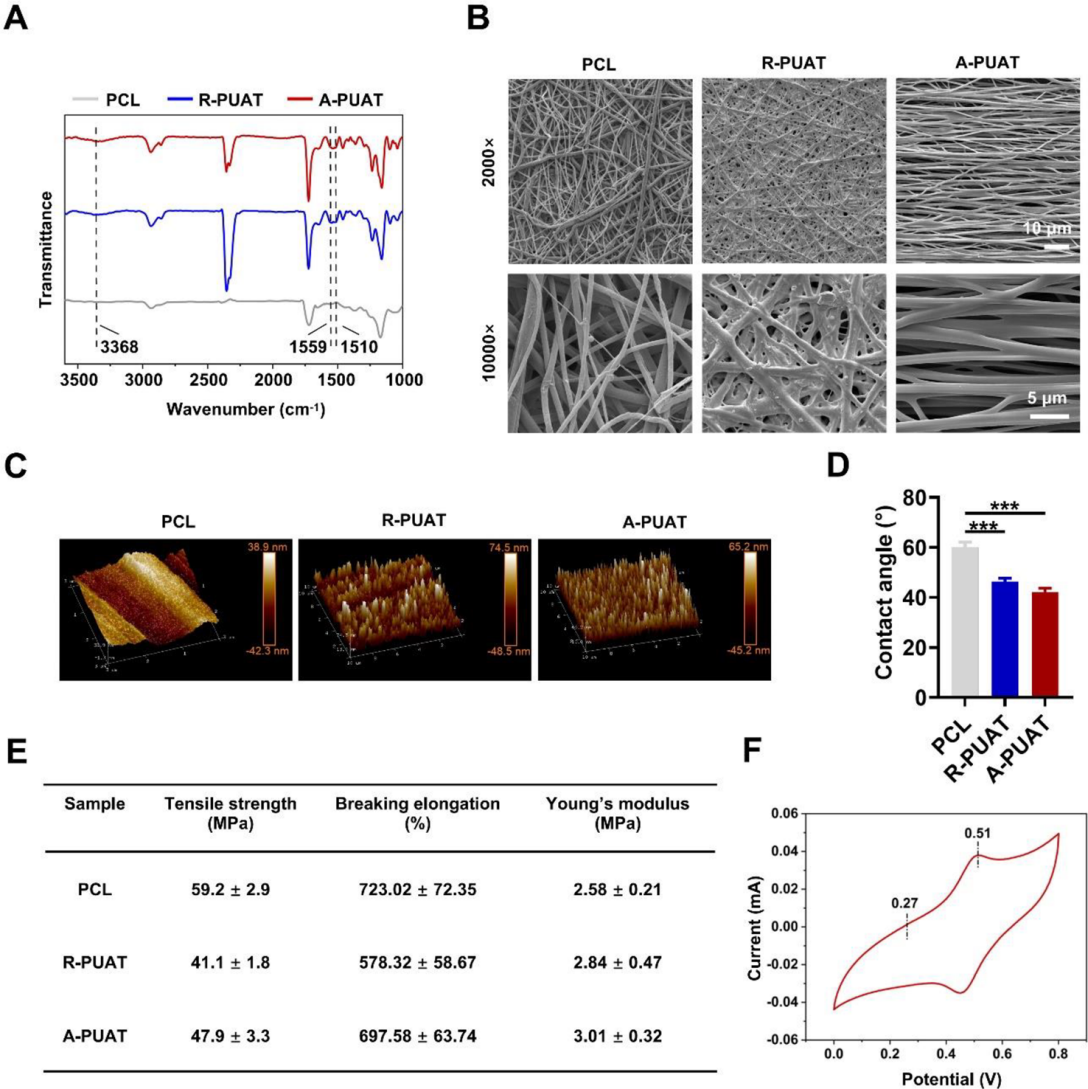
Characterization of PCL, R-PUAT, and A-PUAT membranes. (**A**) FT-IR spectra of each membrane. (**B**) SEM images of each membrane. (**C**) AFM results of each group. (**D**) Water contact angles of each membrane. (**D**) Mechanical properties of each fibrous membrane. (**E**) CV scanning of PUAT polymer in 1 M HCl.

Fig. 1A represented the FT-IR spectra of PCL, R-PUAT and A-PUAT membranes. The characteristic peak appeared at 3368 cm^− 1^ attributed to the vibrational bands of urethane and urea groups. No obvious peak was observed at 2260 cm^− 1^ (N = C stretching) in R-PUAT or A-PUAT, indicating that all –NCO groups of IPDI were completely consumed during the copolymerized process. The characteristic bands of L-lysine (1559 cm^− 1^) and benzene rings of AT (1510 cm^− 1^) were detected in both the PUAT membranes spectra, showing that L-lysine and AT were successfully linked to the PUAT polymer backbone. Surface topography of the PCL, R-PUAT, and A-PUAT was analyzed by SEM and AFM. The fibers were aligned in the A-PUAT membranes but randomly arranged in the PCL and R-PUAT membranes while PUAT polymers increased the roughness of fibrous membranes. (Fig. 1B-C). The average diameters of fibers of PCL, R-PUAT, and A-PUAT membranes were 897.33 ± 155.45 nm, 975.68 ± 133.79 nm, and 908.82 ± 133.67 nm. The water contact angle was measured to determine the hydrophilicity of the different membranes. Comparing to PCL membrane, A-PUAT significantly decreased the water contact angle (Fig. 1D). The mechanical property of each membrane was then analyzed, and the results are shown in Fig. 1E. PUAT membranes exhibited a moderate modulus and a high elasticity, suggesting that they could be sutured to the nerve ends. The PUAT membranes were also resistant to compression from the surrounding soft tissues.

The electroactivity of the PUAT copolymer was verified by CV measurements. In Fig. 1F, two pairs of well-defined reduction/oxidation peaks at about 0.27 and 0.51 V were presented, corresponding to the transitions from leucoemeraldine (LM) to emeraldine state (EM) and emeraldine state to the pernigraniline state (PN) of AT in the PUAT copolymer, respectively. This result indicated the good electroactivity of PUAT copolymer.

### A-PUAT membranes regulate macrophage polarization in vitro

**Fig. 2:**
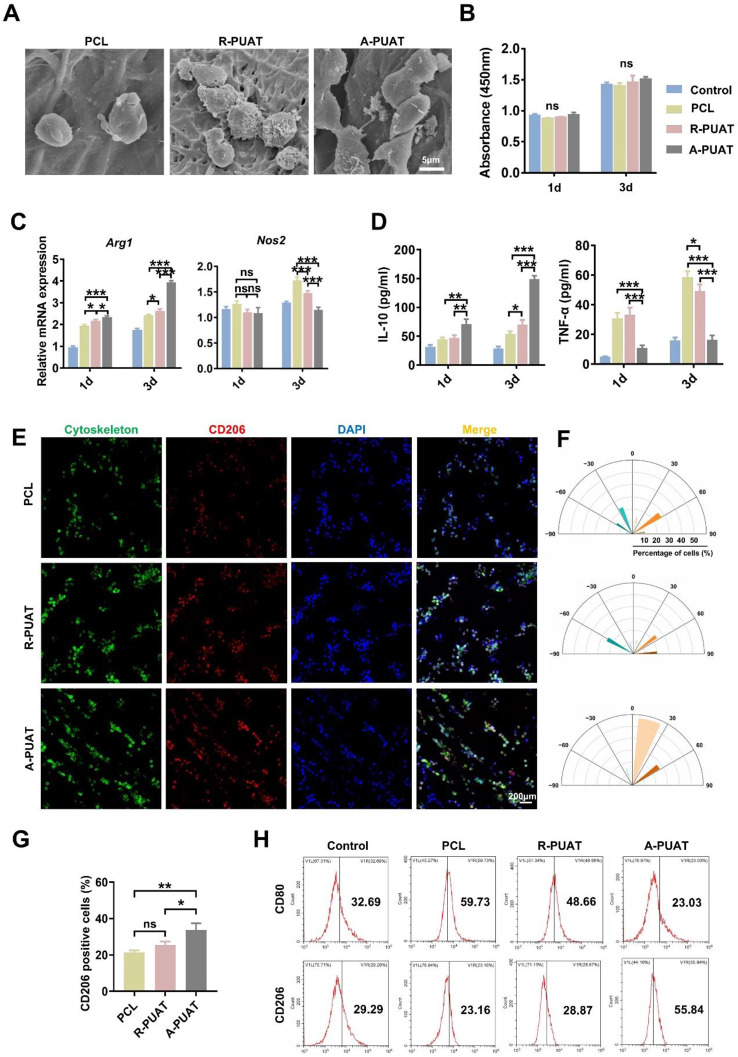
A-PUAT membranes stimulate macrophage polarization to M2-phenotype. (**A**) SEM results revealing the morphology of RAW264.7 cells cultured on different membranes. (**B**) The viability of RAW264.7 cells seeded on each membrane at indicated time points. (**C**) Relative transcription levels of pro-inflammatory gene and anti-inflammatory gene in each group. (**D**) The contents of TNF-α and IL-10 secreted by RAW264.7 cells cocultured on different substrates. (**E**) Representative immunofluorescent images revealing the cytoskeleton of RAW264.7 cells (FITC, green) and M2-phenotype macrophages (CD206, red) after culturing on different membranes for 3 days. (**F**) Quantitative analysis of orientation of macrophages cultured on different membranes. (**G**) Quantitative analysis of CD206 positive cells in RAW264.7 cells seeded on different membranes. (**H**) Flow cytometry showing the expression of CD80 and CD206 in RAW264.7 cells cultured on different substrates.

The morphology of the macrophages on the different membranes was observed by SEM. The macrophages on the PCL and R-PUAT membranes exhibited a spherical/ovoid shape and were randomly stretched out, while the cells on the A-PUAT membranes were elongated and spindle-shaped (Fig. 2A). The CCK-8 assay showed that there was no significant difference in the proliferative activity of cells among the different groups (Fig. 2B). To further evaluate the effect of different membranes on macrophage polarization, RAW264.7 cells cocultured on the different membranes were collected for subsequent analyses. *Nos2* was acknowledged as M1 macrophage marker while *Arg1* was used as M2 macrophage marker. The transcription levels of *Arg1* in both R-PUAT and A-PUAT groups were significantly higher than PCL, while the levels of *Nos2* were markedly lower in PUAT groups (R-PUAT and A-PUAT) than in PCL group (Fig. 2C). Besides, among two PUAT membranes, aligned topography could further increase the expression of *Arg1* and decreased the expression of *Nos2* (Fig. 2C). A similar trend in the cytokine levels of TNF-α and IL-10 was also observed in the ELISA experiments (Fig. 2D). Immunofluorescence analysis revealed that after culturing for 3 days, Raw264.7 cells extended in alignment with the fibers of A-PUAT and showed higher CD206 signal, compared with that seeding on R-PUAT or PCL (Fig. 2E-G). Moreover, flow cytometry detected upregulation of the M2 macrophage marker CD206 and downregulation of the M1 macrophage marker CD80 in the A-PUAT group (Fig. 2H).

**Fig. 3:**
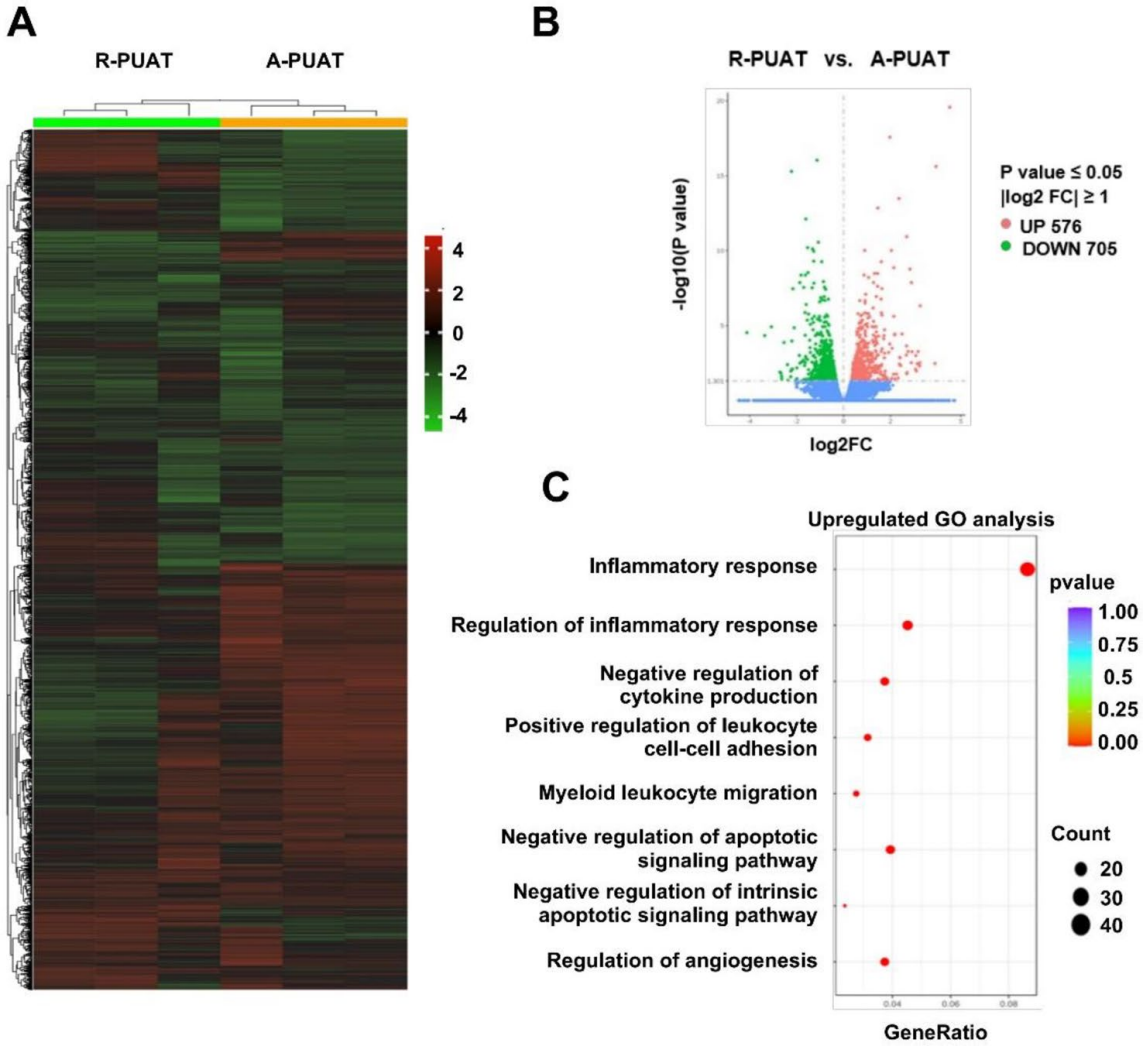
Differentially expressed genes in RAW264.7 cells seeded on R-PUAT and A-PUAT. (**A**, **B**) Heatmap and volcano plot of differentially expressed genes in RAW264.7 cells at 1 day after seeding on different membranes. (**C**) GO analysis of the RNA-sequencing results.

The transcriptomes of RAW264.7 cells seeded on the R-PUAT and A-PUAT membranes were next compared (Fig. 3A). The transcription of 1281 genes changed significantly after seeding on the A-PUAT membranes (|log2fold change|>1, false discovery rate < 0.05). Among them, 576 were upregulated, while 705 were downregulated (Fig. 3B). The GO enrichment analysis indicated that most of the overexpressed genes were associated with regulation of the inflammatory response, cytokine production, cell apoptosis and adhesion, and angiogenesis (Fig. 3C). Collectively, these data indicate that A-PUAT membranes facilitate macrophage polarization toward the M2 phenotype.

### Polarized macrophages stimulating by A-PUAT membranes promoted RSC96 cell migration and myelination in vitro

**Fig. 4:**
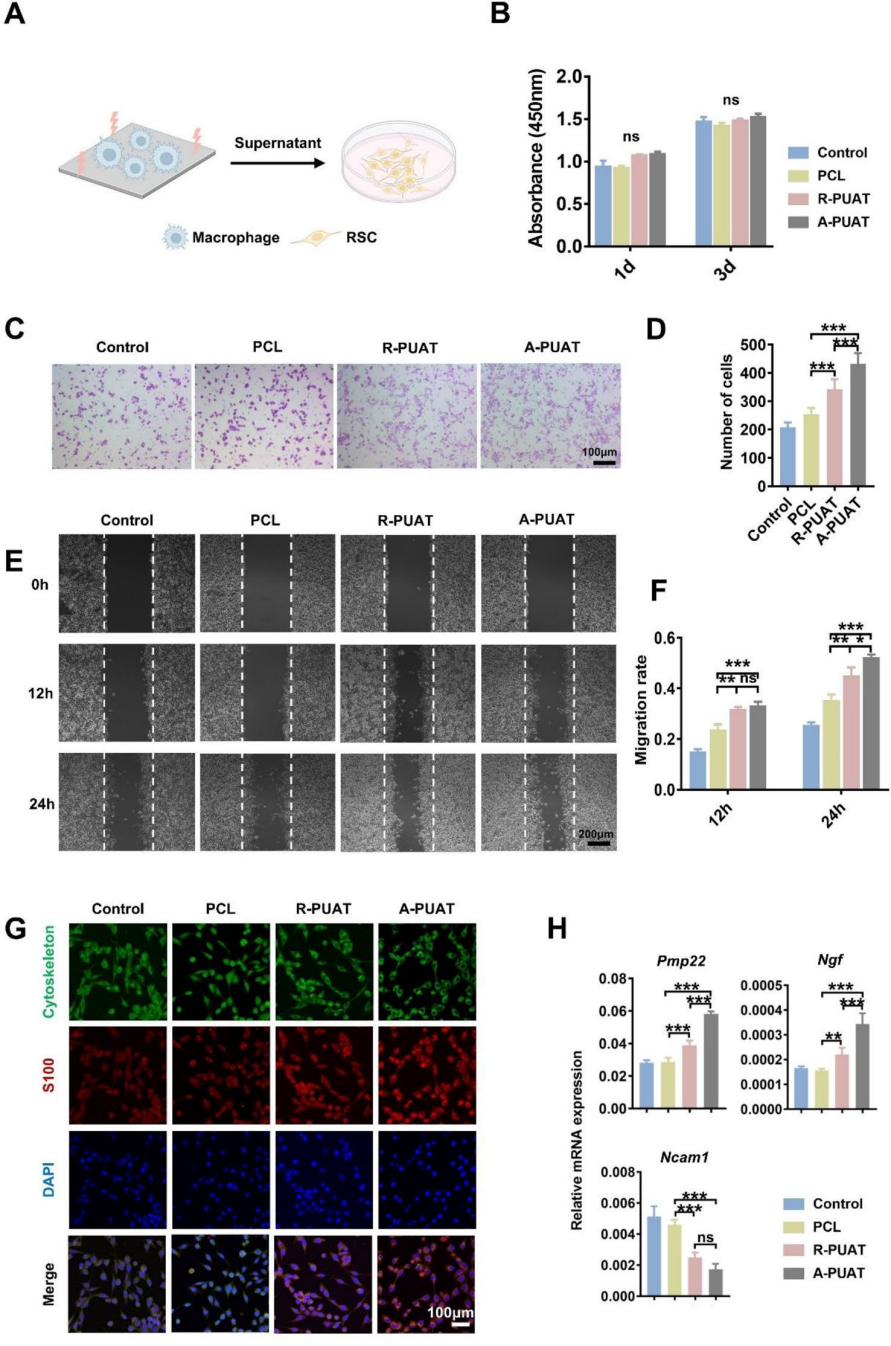
Effects of polarized macrophages induced by different membranes on RSC96 cell behaviors. (**A**) Schematic illustration of RSCs incubated with conditioned medium (CM) from macrophages which were cultured on different membranes. (**B**) The viability of RSCs after cocultured with different CM for 1 or 3 days. (**C**, **D**) Representative images and quantitative analysis of migrated RSCs after cocultured with different CM for 1 day. (**E**, **F**) Representative images and quantitative analysis of the wound healing assay results of RSCs after cocultured with different CM for the indicated time points. (**G**) Representative immunofluorescent images of cytoskeleton (FITC, green) and S100 expression (red) of RSCs cocultured with different CM. (**H**) The transcription levels of myelination-related genes in RSCs after cocultured with different CM for 3 days.

The effects of macrophages cultured on different membranes on the proliferation, migration, and myelination of RSC96 cells were examined (Fig. 4A). There was no significant difference in the proliferative activity of cells among the different groups (Fig. 4B). Transwell assay demonstrated that both R-PUAT and A-PUAT membranes significantly promoted the migration activity of CM-treated RSC96 cells compared with PCL membranes. Besides, comparing to random topography, aligned topography further stimulated the migration of CM-treated RSC96 cells (Fig. 4C-D). Similar tendency was revealed in wound healing assay (Fig. 4E-F). These results suggested that electroactivity of conductive polymer (PUAT) combined with aligned topography could promote migration of RSC96 cells through polarized macrophages. Further immunofluorescent staining showed higher expression of S100β in RSC96 cells cultured with conditioned media which was from A-PUAT-treated macrophages (Fig. 4G). Subsequently, the effect of membranes-stimulated macrophages on myelination of RSC96 cells was detected. qRT-PCR results showed that both two PUAT-based membranes promoted the transcription levels of myelination-related genes peripheral myelin protein 22 (*Pmp22*) and nerve growth factor (*Ngf*) and downregulated the level of neural cell adhesion molecule 1 (*Ncam1*) comparing to PCL group. (Fig. 4H). A-PUAT stimulated the expression of *Pmp22* and *Ngf* compared with R-PUAT (Fig. 4H). The above data indicated that APUAT membranes stimulated migration and myelination of RSC96 cells through modulating macrophage polarization.

### Relevance between macrophages and SCs cells in the early period of grafting

**Fig. 5:**
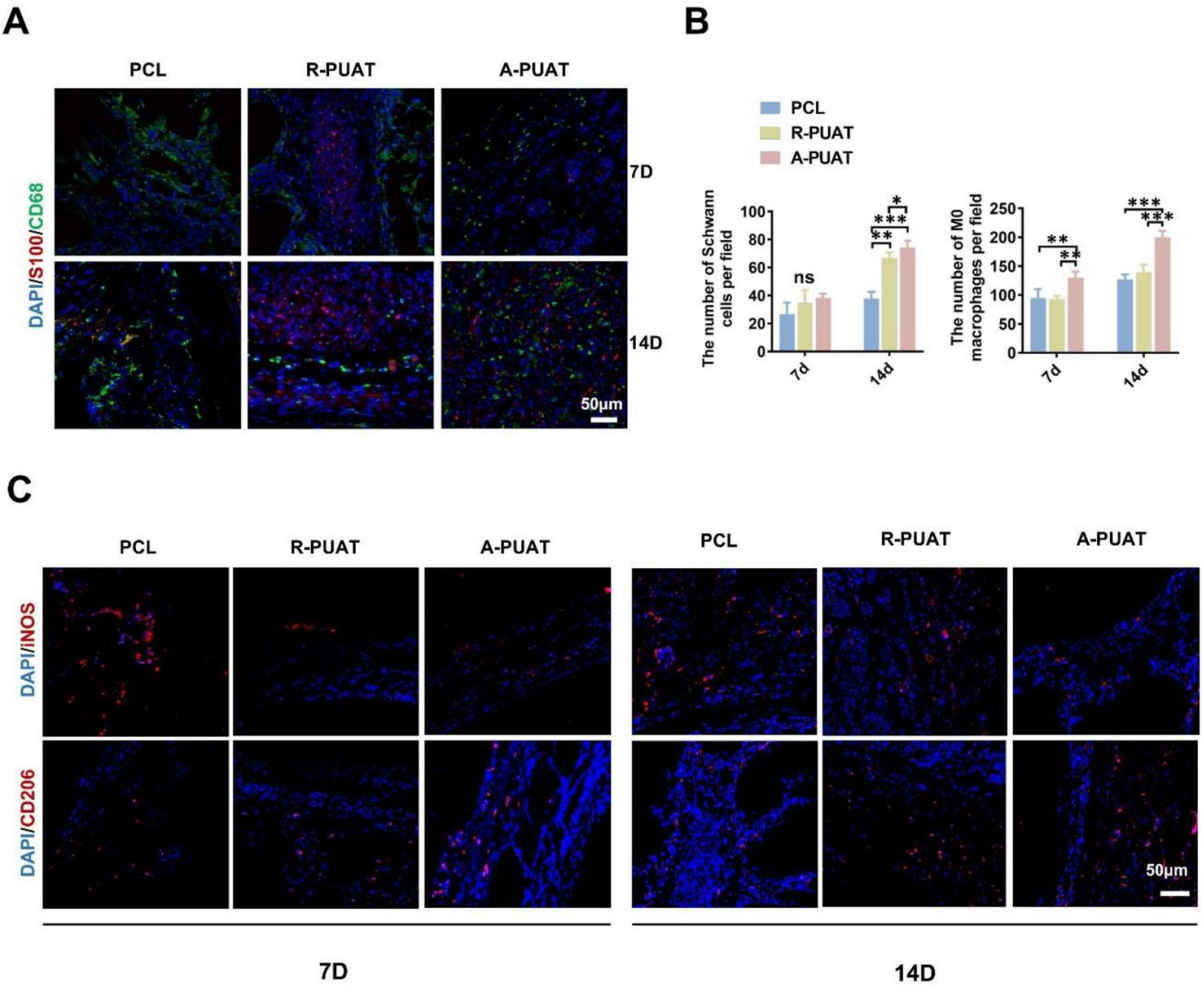
Relevance between macrophages and SCs at 7 and 14 days after grafting. (**A**) Representative immunofluorescence images of the transverse sections of different NGCs showing the distribution of macrophages (M0, CD68, green) and SCs (S100β, red). (**B**) Quantitative analyses showed the number of infiltrated macrophages and SCs in each group. (**C**) Representative immunofluorescence images of M1-phenotype macrophages (iNOS, red), and M2-phenotype macrophages (CD206, red).

The effects of different NGCs on the recruitment of macrophages and SCs were first evaluated at 7 days and 14 days post-operation. The numbers of macrophages notably increased in the regenerated nerves of A-PUAT group at 7 days and 14 days post-operation while SCs markedly increased in A-PUAT group at 14 days after implantation (Fig. 5A-B). Additionally, the polarization of macrophages in regenerated nerves was analyzed by immunofluorescence staining. The A-PUAT group showed the highest percentage of CD206^+^ cells and the lowest percentage of iNOS^+^ cells at both 1 and 2 weeks after operation comparing with other groups (Fig. 5C). These results suggested that A-PUAT NGCs promoted the recruitment of macrophages and stimulated their polarization to the pro-healing M2 phenotype at the early stage of nerve regeneration.

### A-PUAT NGCs promoted the functional recovery of the sciatic nerves

**Fig. 6:**
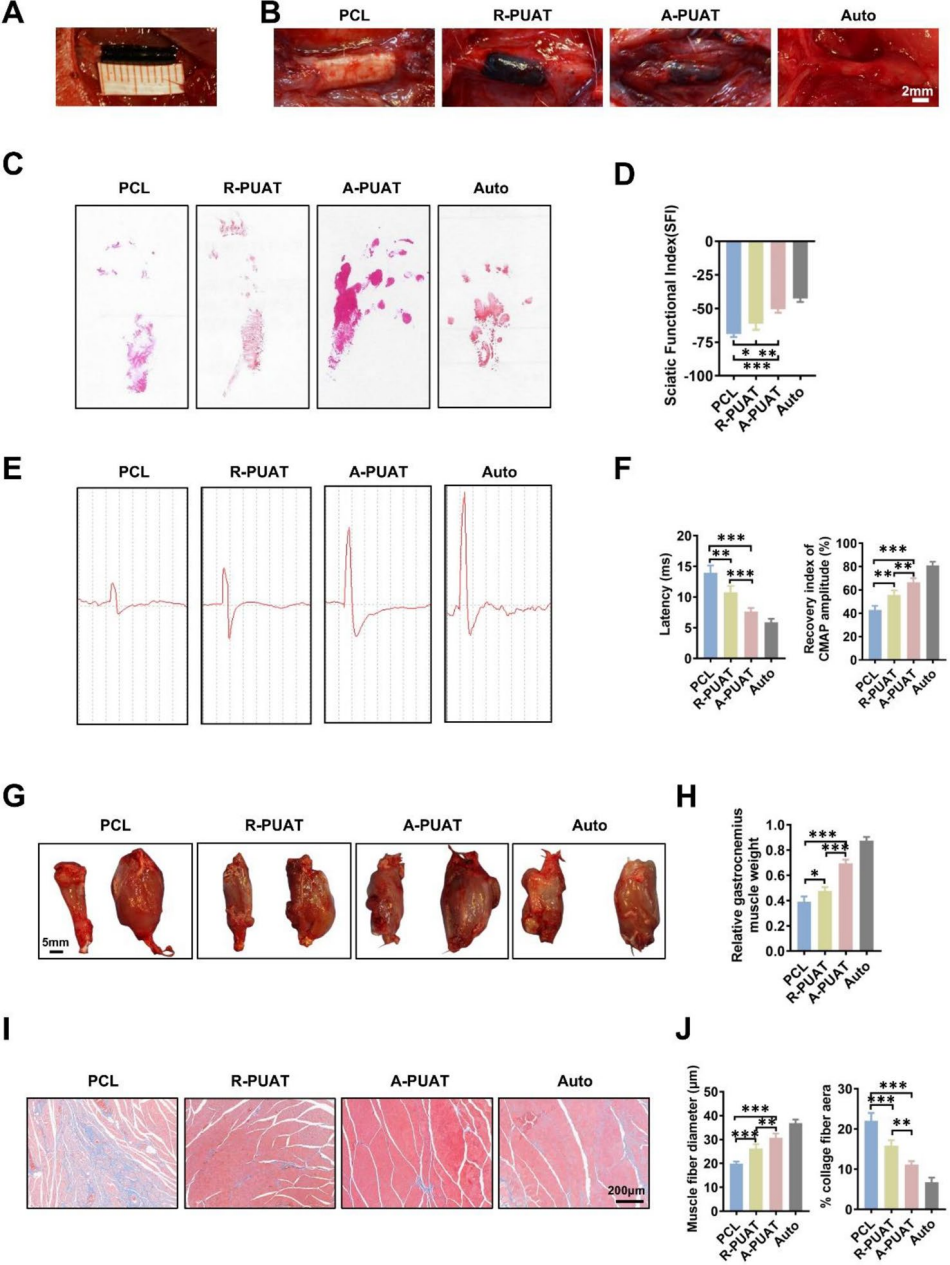
Evaluation of nerve functional recovery at 12 weeks after implantation. (**A**) NGC implantation in a 10-mm long sciatic nerve defect in rat models. (**B**) Gross observation of regenerated sciatic nerves of each group at 12 weeks post-operation. (**C**, **D**) Representative images of footprints and quantitative analysis of the SFI values. (**E**, **F**) Representative CMAP recordings of each group and quantitative analysis of the CMAP amplitude and latency. (**G**) Gross observation of the gastrocnemius muscle in each group at 12 weeks after implantation. The surgery side was on the left, and the contralateral side was on the right. (**H**) Quantitative analysis of the wet weight ratio of the gastrocnemius muscles. (**I**) Representative images of Masson’s staining of the transverse sections of muscles from each group. (**J**) Quantitative analysis of the diameter of muscle fibers and the percentage of collagen-positive area.

To evaluate whether A-PUAT NGCs promoted nerve regeneration in vivo, a rat model of 10 mm-long sciatic nerve injury was established (Fig. 6A), and the effects of each NGC on functional recovery of nerves were evaluated at 12 weeks after grafting. All grafted NGCs were covered with the fascia containing capillaries, and no inflammation was observed (Fig. 6B). The footprints showed a noticeable recovery of the rats in the A-PUAT group, but not those in the PCL group (Fig. 6C). The SFI values of the A-PUAT and Auto groups were slightly different from each other, but both were significantly greater than those of the PCL and R-PUAT groups (Fig. 6D).

An electrophysiological assay was performed to evaluate the functional recovery of the nerves. The conduction latency of the A-PUAT NGCs was significantly shorter than that of the PCL and R-PUAT NGCs, but it was still longer than that of the Auto group. In addition, the CMAP amplitude was comparable between the A-PUAT NGCs and the Auto group, and it was significantly greater than those of the PCL and R-PUAT groups (Fig. 6E-F).

Sciatic nerve regeneration was evaluated by assessing the degree of reinnervation of the gastrocnemius muscle after implantation. Although the muscle weight loss ratio of the A-PUAT group was less than that of the Auto group, it remained significantly greater than those of the remaining groups (Fig. 6G-H). The muscles were then characterized by Masson’s staining. Disordered muscle fibers and collagen deposition were observed in the PCL group, while few fibers were found in the other three groups. The Auto and A-PUAT groups also showed a collagen-positive area that was significantly less than those of the other two groups (Fig. 6I-J).

**Fig. 7:**
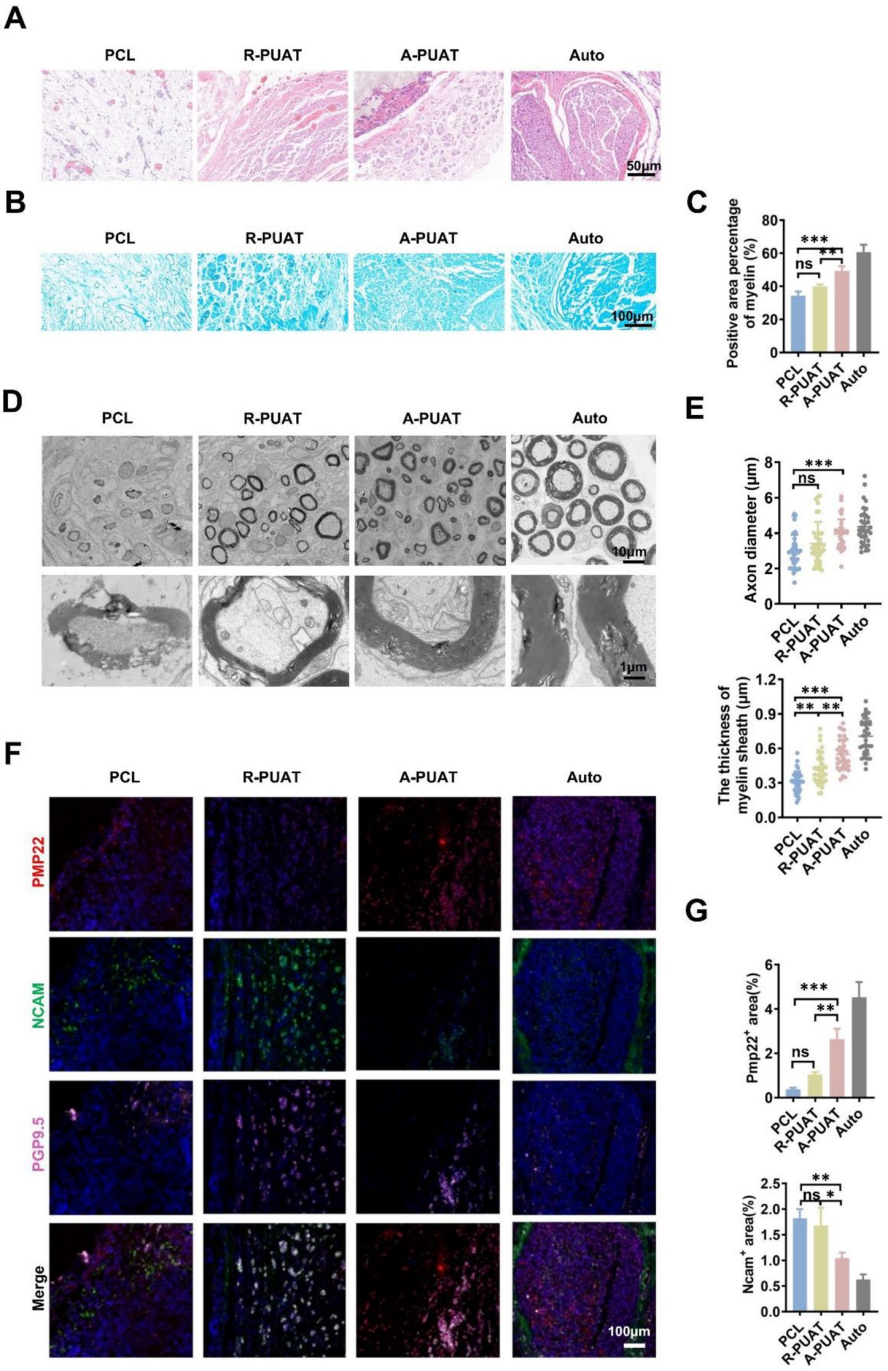
Morphologic and histologic analyses of sciatic nerve regeneration at 12 weeks after implantation. (**A**) Representative H&E staining images. (**B**, **C**) Representative images of Luxol fast blue staining and quantitative analysis of the percentage of myelin-positive area. (**D**, **E**) TEM images of the cross-sections of regenerated nerves and quantitative analysis of the axon diameter and myelin sheath thickness. (**F**, **G**) Representative immunofluorescence images and quantitative analysis of the expression levels of the myelination-related markers PMP22 and NCAM in regenerated nerves.

To analyze the histological and morphological characteristics of regenerated nerves, samples from each group were subjected to H&E staining, LFB staining, and TEM at 12 weeks after implantation. H&E staining detected a large amount of regenerated nerve fibers in the Auto and A-PUAT groups, and no signs of inflammation were observed in any group (Fig. 7A). In addition, LFB staining revealed that the density of myelin was greater in the A-PUAT group than in the R-PUAT and PCL groups, and the largest myelin-positive area was observed in the Auto group (Fig. 7B-C). The TEM images presented the morphology of the regenerated axon fibers. The diameter and thickness of the myelinated nerve fibers in the A-PUAT group were greater than those in the R-PUAT and PCL groups, but they were slightly less than those in the Auto group (Fig. 7D-E).

Lastly, immunofluorescence staining of grafted NGCs was performed to identify the spatial distribution of various types of cells. Compared to the PCL group, the A-PUAT group showed an increased expression of PMP22 and a decreased expression of NCAM surrounding the nerve fibers (Fig. 7F-G). These findings were consistent with the *in-vitro* data, suggesting that A-PUAT NGCs promoted peripheral nerve regeneration and myelination.

## Discussion

**Fig. 8:**
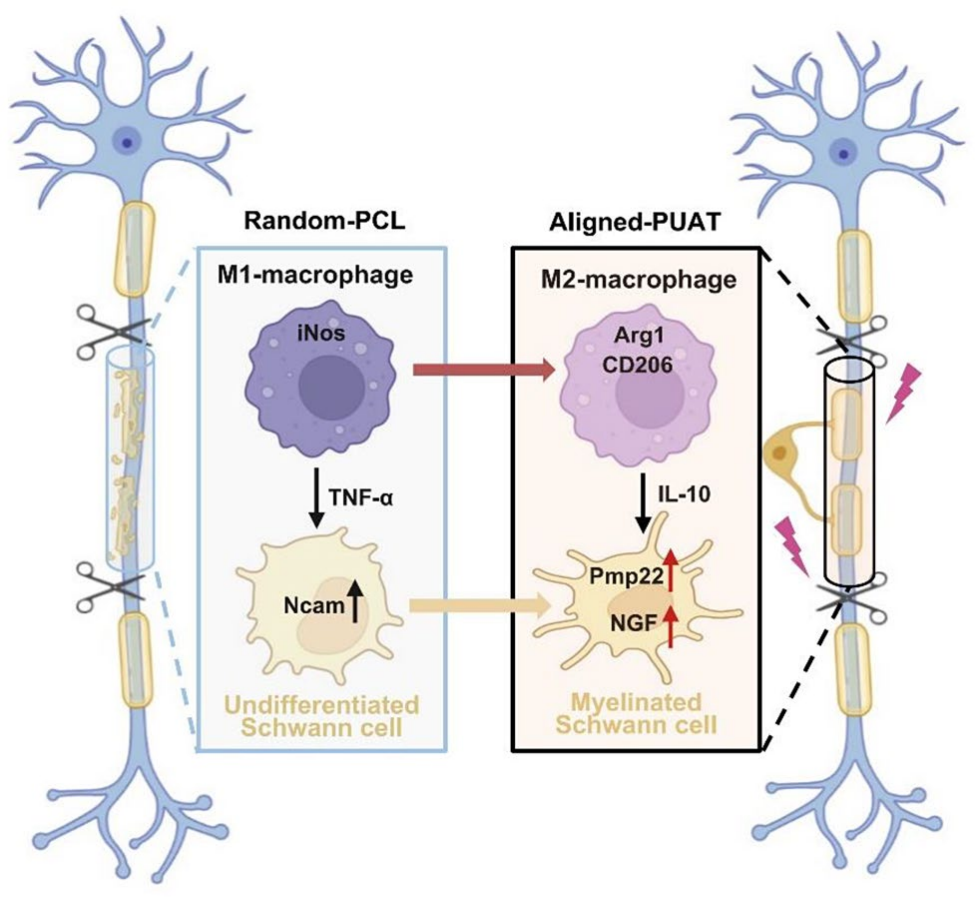
Schematic illustration of nerve regeneration promoted by A-PUAT nerve guidance conduits via modulating macrophage polarization towards the M2 phenotype.

Immunity plays a key role in determining the biocompatibility and modulating the activities of tissue-resident cells; therefore, it affects the outcomes of tissue regeneration [26–31]. Acute and uncontrollable inflammatory responses may impair cell differentiation and nerve regeneration, ultimately leading to implantation failure. However, proper inflammatory responses may promote the recruitment and differentiation of Schwann cells, thus promoting nerve regeneration [15, 32]. The implantation of NGCs after PNI causes activation of the host’s innate immune system, results in significant changes in the local immune microenvironment and therefore affects nerve regeneration. Recent studies of nerve regeneration after PNI have reported delayed regeneration of injured nerves partially caused by inefficient modulation of the regeneration microenvironment [33, 34]. Hence, it is of great importance to create a favorable microenvironment for the regenerative processes using biomaterial-based NGCs [2, 35]. In this study, we designed a novel NGC with conductive polymer and aligned topography and evaluated its effects on immunoregulatory peripheral nerve regeneration both in vitro and in vivo. Our results showed that conductive PUAT NGCs with aligned topography facilitated the recruitment of macrophages and induced their polarization to the pro-healing M2 phenotype, which subsequently enhanced the migration and differentiation of Schwann cells to promote the functional recovery of injured nerves (Fig. 8).

After PNI, Wallerian degeneration exists in the distal end of the nerve injury. Macrophages are recruited to the injuries at early stage and mediate inflammatory response [36]. Macrophages first polarize to M1 phenotype, secrete pro-inflammatory cytokines, and remove myelin debris. When Wallerian degeneration is complete, macrophage will polarize to M2 phenotype, promoting the anti-inflammatory cytokines and achieve nerve regeneration [37]. Macrophages are the most investigated immune component owing to their high plasticity to exogenous stimuli that may affect the regeneration. Previous studies have reported that the immune microenvironment can be affected by the surface topological cues of biomaterials [14, 38, 39]. Porous structures have been proved to influence the polarization of macrophages. The pore size of materials poses limitations for infiltrating cells and ingrowing tissues. 130 μm MAP scaffolds with a pore size that can accommodate 40 μm diameter spheres induce a pro-healing response with early pro-regenerative macrophage profiles, mature collagen regeneration and reduce levels of inflammation in the skin wound [40]. Tina Tylek et al. [41]construct fiber scaffolds with pore sizes between 40 and 100 μm. These scaffolds lead to elongation of primary human macrophages, accompanied by a polarization towards the M2 phenotype. Direct regulation of the shape of macrophages to an elongated state by micropatterning methods can promote macrophages polarize to M2 phenotype and reduce the synthesis of proinflammatory cytokines [42]. Aligned electrospun nanofibers can suppress the M1 macrophage phenotype and inhibit inflammatory progression via JAK-STAT and NF-κB signaling pathways [43]. Consistent with these studies, our results revealed that macrophages cocultured on A-PUAT membranes elongated, and polarized to M2 phenotype, suggesting that modifying the topological cues may be a promising strategy for immunomodulation.

As a topological cue, the arrangement of nerve fibers can also influence cell migration and rearrangement to accelerate axon regeneration [44]. Biomimetic NGCs constructed by aligned fibers with highly integrated functionalities are beneficial for tissue regeneration because they can guide the direction of cell growth and migration to the injured sites [4, 12–14]. Manasa Nune et al. [45] have proved that self-assembling peptide nanostructures on aligned PLGA nanofibers can provide better cell–matrix communication and stimulate nerve remyelination. Other study indicates that uniaxially oriented fibers could guide migration of SCs but surface properties of scaffolds may be more influential than scaffold morphology in nerve regeneration [46].

Desirable mechanical properties of NGCs are expected to recreate a natural ECM-like physical structure and mechanical strength for neural repair [47]. Our previous study has demonstrated the biocompatibility and high flexibility of conductive PUAT [22]. In the present study, we fabricated PUAT-electrospun membranes with alignment topography and found that the uniform orientation of nanofibers strengthened the mechanical properties of membranes. Additionally, differential densities and sizes of porous structures may also contribute to the mechanical property regulation of NGCs [48]. Radial compression of NGCs may occur through muscular movements [49], and proper porosity of NGCs provides mechanical strength of fixation and guarantee sufficient oxygen supply and nutrients for nerve regeneration [48].

The regeneration of complicated nerve structures requires the proliferation and coordination of various types of cells over a long distance [50, 51]. Before nerve axon regeneration, a “bridge” should be built between the stumps of the injured nerves to guide axon regeneration, which requires the migration of macrophages, SCs, fibroblasts, and endothelial cells. Our results revealed that polarization of macrophages into the M2 phenotype by A-PUAT membranes could promote migration of SCs, the expression of S100β, and the transcription levels of myelination-related genes, strengthening the idea of promoting peripheral nerve regeneration by modulation of macrophages. Future studies may focus on the molecular mechanisms that how M2 macrophage modulates SCs.

Neural tissue consists of nerve bundles with multiple arranged components that transmit information primarily through action potentials generated by synapses [52]. Therefore, the orientation and conductivity of the matrix are of great benefit in guiding the contact of neurons and the transmission of electrical signals. Electrical signals are not only the information transmitted by neurons, but also play a role in regulating nerve growth as well as other neurophysiological activities. The density of nerve fibers increases with electrical stimulation, which in turn increases nerve function. Appropriate electrical stimulation can promote nerve growth and inhibit the inflammatory response. Previous study constructed a PCL-GO nanofibrous scaffolds coated with acellular matrix, the antioxidant activity and electroactivity of GO stimulated remyelination for facilitating nerve regeneration [53]. In addition, melanin incorporated electroactive and antioxidant silk fibroin nanofibrous scaffolds promoted the neuronal differentiation through antioxidant and radical scavenging properties [54]. Electrical signals are also proved to regulate macrophage polarization, phagocytosis activity and cytokine production. Yao et al. [55] fabricated rGO@UIO-66/PCL nerve conduits, and the electroactive interface could affect inflammatory and metabolic signaling pathways that promoted nerve regeneration. Conductive polymers have shown promising applications in nerve tissue engineering due to their unique combination of electrical conductivity and biocompatibility [56]. In our study, conductive PUAT polymer was utilized for fabricating electrospun membranes. The electrical conductivity of PUAT contributed to macrophage polarization and thus myelination of SCs to achieve nerve regeneration.

Although the potential use of A-PUAT NGCs in PNI has been proposed, hollow NGCs are less effective than autografts in functional recovery and bridging long gaps [57, 58]. Further investigations on the supportive intraluminal guiding structure that can simulate the structural patterns of the endoneurium are needed for better nerve regeneration. In addition, local immune responses following biomaterial implantation may not be limited to the activation of macrophages [29, 31, 59–61]. Monocyte immunomodulation also plays an important role in tissue regeneration and angiogenesis. Future studies on other types of immune cells, such as neutrophils and T cells, and their contributions to nerve regeneration are warranted.

## Conclusion

In this study, an aligned conductive nanofibrous NGC was fabricated using PUAT polymer (A-PUAT). A-PUAT NGC facilitated the recruitment of macrophages and induced macrophage polarization to the pro-healing M2 phenotype. Subsequently, macrophages polarized by the A-PUAT NGC promoted the migration and myelination of SCs both in vitro and in vivo. Our study suggested A-PUAT NGC which provides electrophysiological and topological signals as an ideal conductive biomaterial to modulate immune microenvironment for promoting peripheral nerve regeneration.

## Data Availability

No datasets were generated or analysed during the current study.
